# Psychic Power and Boxing

**Published:** 1920-02-14

**Authors:** 


### Psychic Power and Boxing.

The staff of the Neurological War Hospital have made many valuable scientific contributions during the past year, but we have read none which has surpassed in interest the remarkable essay by Dr. Arthur Hadfield in a new book called "The Spirit," edited by Canon Streeter, and published by Macmillan. A series of experiments are described whereby the influence of suggestion on physical strength are demonstrated. Three men, one of whom was a prize-fighter, submitted themselves to test the effect of mental suggestion on their strength, which was measured by gripping a dynamometer. At the time of experiment the men were in their normal waking condition and were tested (*a*) after suggesting to them under hypnosis that they were "weak"; (*b*) after suggesting under hypnosis that they were very strong.

The dynamometer was held in each case as tightly as possible—that is to say, the will power was extended to the utmost—and was found to register an average grip of 101 lb. in the normal waking condition; whilst under hypnosis and under the suggestion that they were "very weak" only 29 lb. was registered, and when "strength" was suggested as much as 142 lb. was indicated by the instrument. It is assumed that when "weakness" was suggested the full flood of energy was checked, and the men were capable of only one-third of their normal strength, whereas by suggestion of "strength" latent powers were liberated and their usual or normal strength increased by half as much again. Dr. Hadfield concludes from this experiment that the limits of possibility in our daily lives, and the resources of power are psychic rather than physical in character.

At the present time Georges Carpentier and Jimmy Wilde, both men of comparatively frail physique, are champions of the boxing art.

Dr. Hadfield's experiments provide a possible solution to the secret of their continued success.

